# Phase-change properties of GeSbTe thin films deposited by plasma-enchanced atomic layer depositon

**DOI:** 10.1186/s11671-015-0815-5

**Published:** 2015-02-28

**Authors:** Sannian Song, Dongning Yao, Zhitang Song, Lina Gao, Zhonghua Zhang, Le Li, Lanlan Shen, Liangcai Wu, Bo Liu, Yan Cheng, Songlin Feng

**Affiliations:** State Key Laboratory of Functional Materials for Informatics, Shanghai Institute of Micro-system and Information Technology, Chinese Academy of Sciences, Shanghai, 200050 China; Division of Nuclear Materials Science and Engineering, Shanghai Institute of Applied Physics, Chinese Academy of Sciences, Shanghai, 201800 China

**Keywords:** Phase-change memory, Atomic layer deposition, Microstructure, Electric properties

## Abstract

Phase-change access memory (PCM) appears to be the strongest candidate for next-generation high-density nonvolatile memory. The fabrication of ultrahigh-density PCM depends heavily on the thin-film growth technique for the phase-changing chalcogenide material. In this study, Ge_2_Sb_2_Te_5_ (GST) and GeSb_8_Te thin films were deposited by plasma-enhanced atomic layer deposition (ALD) method using Ge [(CH_3_)_2_ N]_4_, Sb [(CH_3_)_2_ N]_3_, Te(C_4_H_9_)_2_ as precursors and plasma-activated H_2_ gas as reducing agent of the metallorganic precursors. Compared with GST-based device, GeSb_8_Te-based device exhibits a faster switching speed and reduced reset voltage, which is attributed to the growth-dominated crystallization mechanism of the Sb-rich GeSb_8_Te films. These results show that ALD is an attractive method for preparation of phase-change materials.

## Background

Phase-change memory (PCM) has been regarded as one of the most promising nonvolatile memories for the next generation because of the advantages of high speed, low power, good endurance, high scalability, and fabrication compatibility with complementary metal-oxide semiconductor (CMOS) process [1-4]. PCM uses the reversible phase change between the crystalline and amorphous states of chalcogenide materials brought about by Joule heating. Ternary Ge_2_Sb_2_Te_5_ (GST) compounds are widely regarded as the most commercially viable and practical phase-change family of materials for this application. These materials are currently being used commercially, and processes which deposit GST films by RF sputtering are being implemented into production lines [5].

In PCM cells, the high level of reset current, which is required for switching the GST material from the crystalline to the amorphous state in a planar cell structure, has been the major obstacle to the further scaling of PCM because of the limited on-current drive capability of the cell transistor [6]. It has been reported that a confined cell structure where the phase-change material is formed inside a contact via is expected to be essential for the next-generation PCM device because it requires lower switching power [7,8]. This structure requires more complex deposition of the active chalcogenide into a very small cell pore. However, it can be easily anticipated that the fabrication of this confined structure is not possible using the conventional sputtering process for the GST film deposition due to its inherent poor step coverage. Therefore, it is necessary to deposit the GST film using a process that offers good conformality in terms of its thickness as well as its chemical composition, such as atomic layer deposition (ALD) or chemical vapor deposition (CVD).

Recently, a number of studies have been carried out to investigate the deposition of phase-change materials by ALD and CVD techniques [9-15]. It has been reported that the precursor and substrates have an important influence on the microstructure and composition of Ge-Sb-Te films. Mikko Ritala *et al*. reported Sb_2_Te_3_, GeTe, and GST films were deposited by ALD at remarkably low temperature of 90°C using (Et_3_Si)_2_Te, SbCl_3_, and GeCl_2_ · C_4_H_8_O_2_ as precursors [10]. Byung Joon Choi *et al*. reported the different nucleation and growth behaviors of the GST films deposited by the combined plasma-enhanced CVD/ALD on various types of substrates. The nucleation of the GST films on the SiO_2_, Si_3_N_4_, and ZrO_2_ substrates was seriously retarded compared to those on the TiN and TiO_2_ substrates [11]. Adulfas Abrutis *et al*. reported the deposition of smooth GST films by using a hot-wire CVD technique [12]. Most of these works focus on the microstructure properties and the deposition process. However, the electric properties and switching properties of ALD/CVD-deposited phase-change materials (especially Sb-rich GeSbTe films) have rarely been reported in literature. In this study, ALD of Sb_2_Te_3_, GST, and GeSb_8_Te thin films were attempted with Ge [(CH_3_)_2_ N]_4_, Sb [(CH_3_)_2_ N]_3_, Te(C_4_H_9_)_2_ as Ge, Sb, and Te precursors, respectively, and plasma-activated H_2_ gas as reducing agent of the metallorganic precursors. The microstructural and electric properties of these materials have been studied.

## Methods

The Sb_2_Te_3_ and Ge-Sb-Te films were deposited on Si_3_N_4_/Si substrates using a plasma-enhanced ALD reactor (Beneq TFS 500 ALD system) at wafer temperatures ranging from 190 to 250°C. The Si_3_N_4_ films were prepared by inductively coupled plasma CVD on Si substrate at temperature of 130°C. The SiH_4_ and NH_3_ were used as reactive gases. Ge [(CH_3_)_2_ N]_4_, Sb [(CH_3_)_2_ N]_3_, and Te(C_4_H_9_)_2_ were used as the Ge, Sb, and Te precursors, respectively. Each precursor deposition cycle for the Ge, Sb, and Te process was composed of four consecutive pulses: (i) a pulse of precursor vapor, (ii) a purge pulse, (iii) a pulse for exposure to H_2_ plasma, and (iv) another purge pulse. The sequence of precursor pulses was Sb-Te-Ge. To control the cation composition ratio in Ge-Sb-Te films, the pulse ratio of precursor cycles was changed. During the H_2_ plasma pulse period, a radio frequency (rf) plasma was applied (rf power of 150 W, rf frequency of 13.56 MHz) to the reaction chamber. The growth rates of GST and Sb_2_Te_3_ films are about 0.2 and 0.1 nm/cycle, respectively. The uniformity of Sb_2_Te_3_ films was very good, but the uniformity of GST was very sensitive to the deposition process. Similar results have been found by other researchers [13]. The stoichiometry of the deposited films was confirmed by electron dispersive spectroscopy (EDS). The thickness and microstructure of the films were determined by field-emission scanning electron microscopy (FESEM). T-shaped PCM cells are fabricated using 0.18 μm CMOS technology to verify the electrical switching behaviors of the alloys. The bottom W electrode with diameter of 260 nm is covered by phase-change films of approximately 100 nm thickness, while TiN (20 nm) and Al (300 nm) are deposited sequentially as top electrodes. For comparison, physical vapor deposition (PVD) GST film was also fabricated with the same structure. The current–voltage (*I-V*) and resistance-voltage (*R-V*) characteristics of the PCM cells are monitored employing an arbitrary waveform generator (Tektronix AWG5002B) and a Keithley-2400 meter.

## Results and discussion

**Figure 1:**
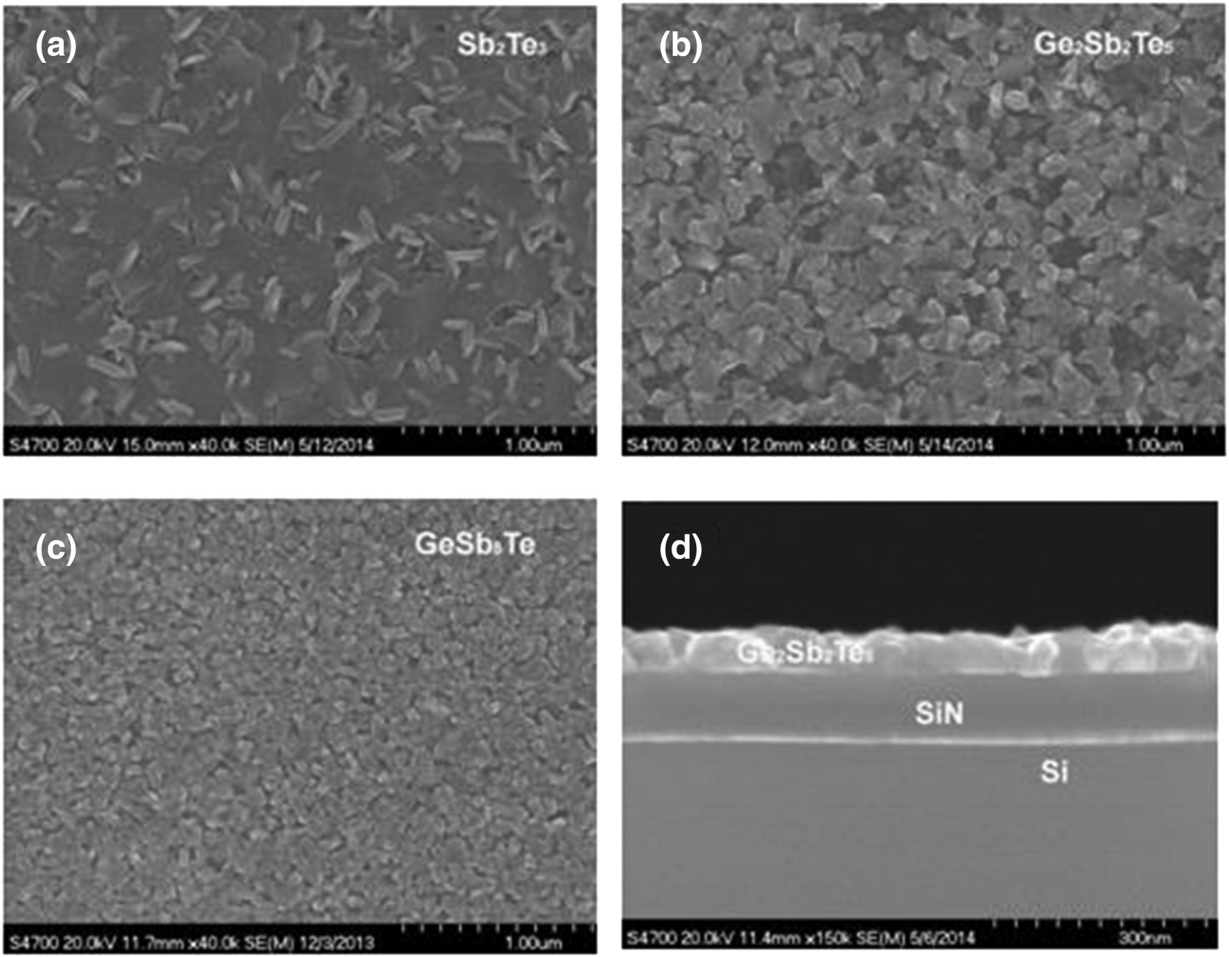
**SEM surface micrographs.** SEM surface micrographs of **(a)** a 70-nm Sb2Te3 film grown on Si3N4, **(b)** a 60-nm Ge2Sb2Te5 film grown on Si3N4, **(c)** a 70-nm GeSb8Te film grown on Si3N4, **(d)** cross-section structure of the 60-nm Ge2Sb2Te5 film grown on Si3N4.

**Figure 2:**
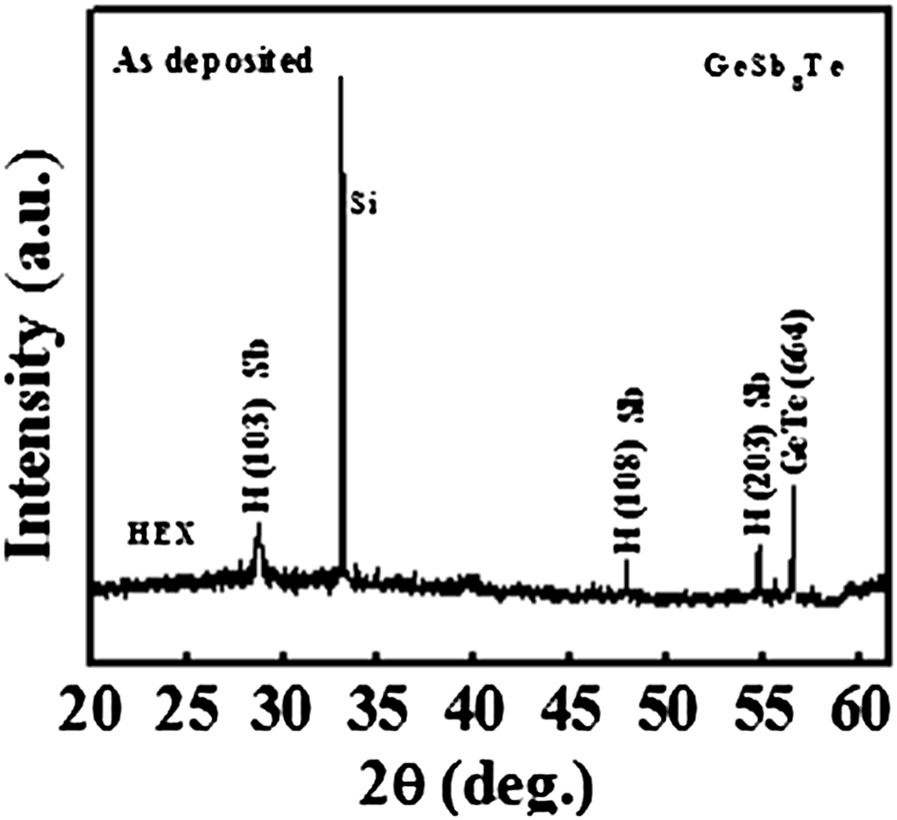
**XRD patterns of GeSb**_**8**_ **Te thin film deposited on Si**_**3**_ **N**_**4**_ **/Si substrate at 200°** **C.**

Figure 1a to d shows the SEM surface micrographs of a 70-nm Sb_2_Te_3_ film, a 60-nm GST film, a 70-nm GeSb_8_Te film, and the cross-section structure of the 60-nm GST film grown on Si_3_N_4_ surfaces, respectively. It can be seen in the figure that pure Sb_2_Te_3_ film shows much larger crystal grain size than those of the GST and GeSb_8_Te films. The GST film grown on a Si_3_N_4_ surface appeared to have many surface voids. The grain size of GST film is about 100 nm. The GeSb_8_Te film grown on a Si_3_N_4_ surface showed a similar surface morphology with a slightly smaller average grain size (approximately 70 nm) and without surface voids. The root-mean-square (rms) surface roughness values of Sb_2_Te_3_, GST, and GeSb_8_Te are 43.5, 30.4, and 21.8 nm, respectively, indicating rough surface morphology of these films. The cross-sectional image in Figure 1d confirms the rough surface morphology of the GST film. XRD patterns of GeSb_8_Te thin film deposited on Si_3_N_4_/Si substrate at substrate temperature of 200°C are shown in Figure 2. Hexagonal Sb and additional GeTe phases were seen in GeSb_8_Te film, which indicates that it crystallized during deposition. This is due to the low crystallization temperature (<200°C) of the GeSb_8_Te film.

**Figure 3:**
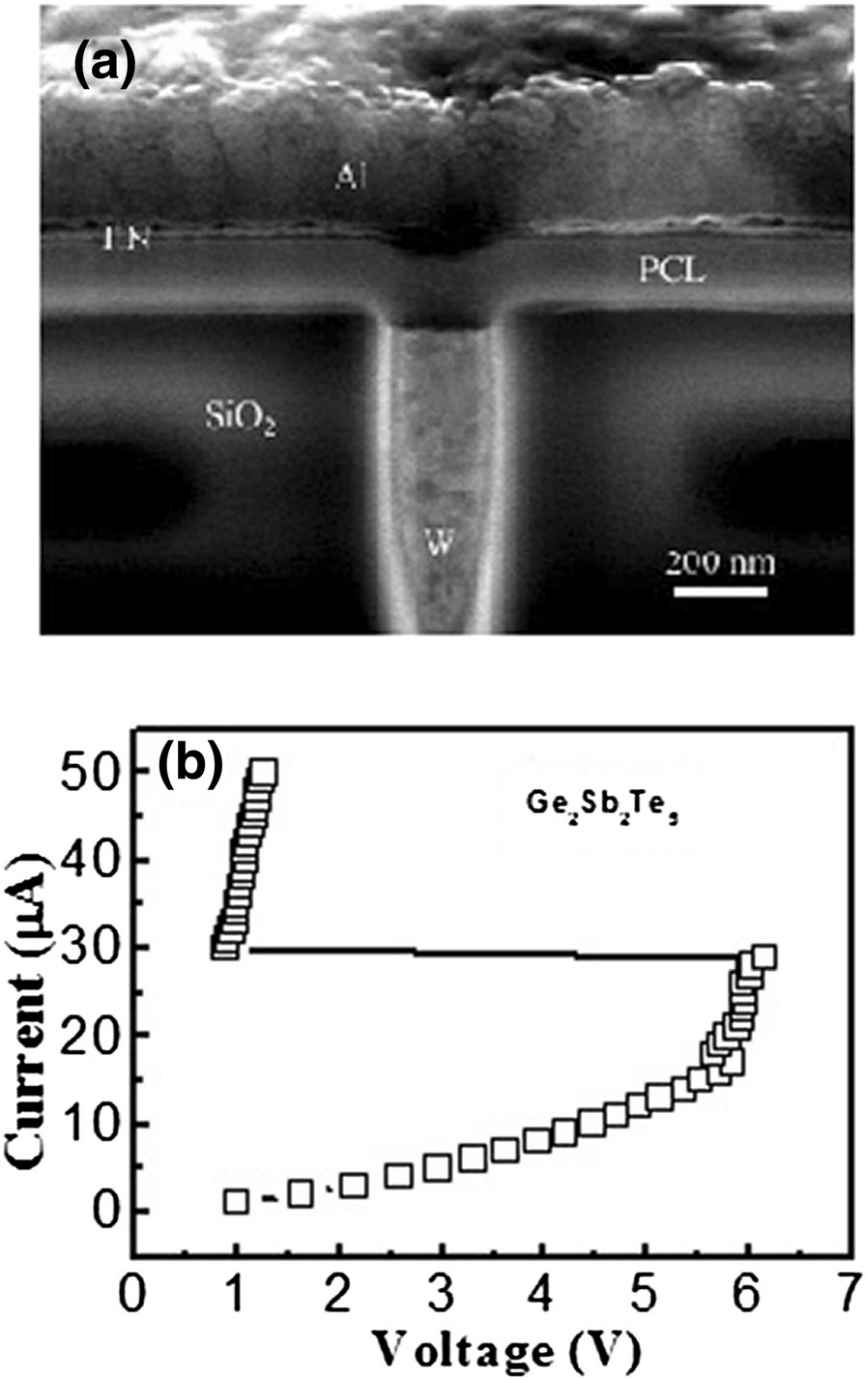
**Device structure for a single cell and typical** ***I*** **-** ***V*** **curves of the PCM cells. (a)** SEM image of cross-sectional cell structure. **(b)** Resistance current characteristics of PCM cell with ALD-deposited GST films.

For further investigation of device performances, GST and GeSb_8_Te films were selected and inserted in PCM cells. The device structure for a single cell is shown in Figure 3a. Figure 3b shows the typical *I*-*V* curves of the PCM cells based on ALD-deposited GST films. Before each *I*-*V* test, the cell has been re-amorphized using electrical pulse. As shown in Figure 3b, large snapback of voltage and negative-resistance behavior were observed in the *I*-*V* curve, which indicates that the phase transition has occurred from amorphous state (high resistance) to crystalline state (low resistance). The threshold voltage for the cell based on ALD-deposited GST is about 6.1 V, which is much higher than that (3.5 V) of the device based on PVD-deposited GST with the identical cell architecture [16].

**Figure 4:**
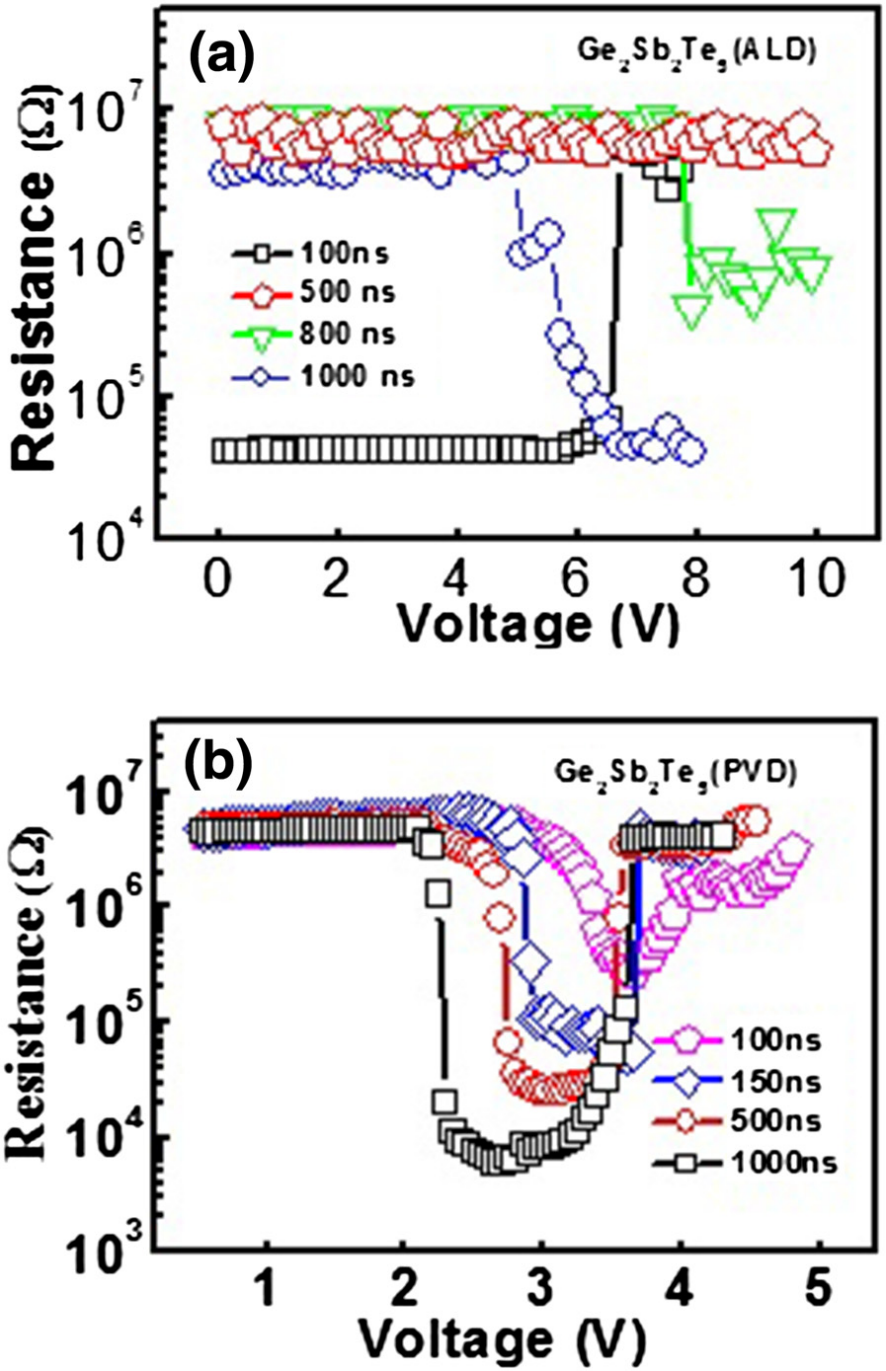
**Resistance voltage characteristics of PCM cell ((a) ALD-deposited GST, (b) PVD-deposited GST films by different voltage pulse widths).**

The phase transition of PCM cell can be characterized from the relation between the cell resistance and the corresponding amplitude of voltage pulse or current pulse (so-called *R-V* or *R-I* curve). In order to test the electrical phase-change ability of the ALD-deposited GST films, the SET and RESET operations of the PCM cells based on the ALD-deposited GST materials are realized by a stimulated voltage pulse with different pulse widths, as presented in Figure 4a. As indicated in Figure 4a, the resistance dramatically increases by two orders of magnitude at the reset voltage of around 6.7 V. The *R-V* curves of the PCM cells using PVD-deposited GST materials are also shown in Figure 4b. For the device based on PVD-deposited GST films with the identical cell architecture, the reset voltage is around 3.6 V, which is lower than that of the device based on ALD-deposited GST. It also can be seen from Figure 4b that a 200-ns-wide voltage pulse is able to set the cells based on PVD-deposited GST materials with the sensing margin (*R*_RESET_/*R*_SET_) of more than two orders of magnitude. For the device based on ALD-deposited GST, it is noted that a 500-ns-wide pulse fails to set the cell and a pulse width of 800 ns is insufficient for a complete set programming, suggesting that ALD process indeed leads to a slower crystallization process thus longer write time for the set operation. The measured set speed of the ALD-deposited GST films is slower than that of PVD-deposited GST films, which is supposedly due to the impurities. Especially N, C impurities in ALD-deposited GST films are known to increase the crystallization temperature and reduce the crystallization speed of the GST films [17,18].

**Figure 5:**
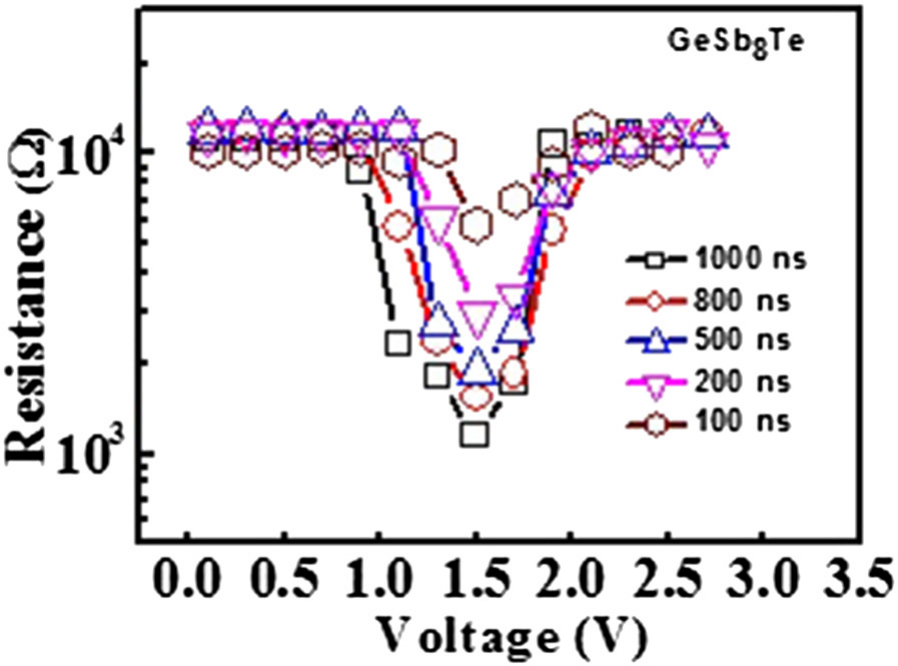
**Resistance voltage characteristics of PCM cell with ALD-deposited GeSb**_**8**_ **Te film by different voltage pulse widths.**

The *R-V* curves of the PCM cells based on ALD-deposited GeSb_8_Te material with various pulse widths are plotted in Figure 5. Reversible phase-change process has been observed. As revealed, once the programing voltage increases beyond the threshold voltage, the cell resistance starts to drop due to the crystallization of GST alloy and then reaches a minimum, which is corresponding to the set resistance. When the voltage is further increased, the resistance again rises and then returns to the reset state. The minimum set and reset voltages decrease with the increasing pulse width due to the equivalent energy required to crystallize and melt the programming region, respectively. The set resistance of the cells decreases with the pulse width, obtaining a lower resistance of approximately 1.1 kΩ for the 1,000-ns-wide pulse, which is attributed to the larger grains and/or a more complete crystallization state. As indicated in the figure, the reset voltage of approximately 2.1 V of the cell is clearly lower than that of the ALD GST-based cells (approximately 6.7 V) and PVD GST-based cells (approximately 3.7 V) with the identical cell architecture. The resistance window could be obtained by an electric pulse as short as 100 ns. Compared with ALD GST requiring 1,000 ns in this study, GeSb_8_Te exhibits a faster switching speed of more than 10 times. The growth-dominated crystallization mechanism of the Sb-rich GeSb_8_Te film could account for its ultra-fast speed, while GST requires much more time for nucleation due to its nucleation-dominated crystallization mechanism.

## Conclusions

In this study, GST and GeSb_8_Te thin films were deposited by plasma-enhanced ALD method using Ge [(CH_3_)_2_ N]_4_, Sb [(CH_3_)_2_ N]_3_, Te(C_4_H_9_)_2_ as precursors and plasma-activated H_2_ gas as reducing agent of the metallorganic precursors. The measured set speed of the ALD-deposited GST films is slower than that of PVD-deposited GST films, which is supposedly due to the N, C impurities. Compared with GST-based device, GeSb_8_Te-based device exhibits a faster switching speed and reduced reset voltage, which is attributed to the growth-dominated crystallization mechanism of the Sb-rich GeSb_8_Te films. These results show that ALD is an attractive method for preparation of phase-change materials.
